# Estimating Kidney Function in the Critically Ill Patients

**DOI:** 10.1155/2013/721810

**Published:** 2013-05-08

**Authors:** Gemma Seller-Pérez, Manuel E. Herrera-Gutiérrez, Javier Maynar-Moliner, José A. Sánchez-Izquierdo-Riera, Anibal Marinho, José Luis do Pico

**Affiliations:** ^1^Department of Critical Care Medicine, University Hospital Carlos Haya, 29018 Malaga, Spain; ^2^University of Malaga School of Medicine, Spain; ^3^Department of Critical Care Medicine, Santiago Hospital, Vitoria, Spain; ^4^Department of Critical Care Medicine, Hospital 12 de Octubre, Madrid, Spain; ^5^Department of Critical Care Medicine, Centro Hospitalario de Porto, Portugal; ^6^Department of Critical Care Medicine, Hospital Municipal de Necochea, Argentina

## Abstract

Glomerular filtration rate (GFR) is an accepted measure for assessment of kidney function. For the critically ill patient, creatinine clearance is the method of reference for the estimation of the GFR, although this is often not measured but estimated by equations (i.e., Cockroft-Gault or MDRD) not well suited for the critically ill patient. Functional evaluation of the kidney rests in serum creatinine (Crs) that is subjected to multiple external factors, especially relevant overhydration and loss of muscle mass. The laboratory method used introduces variations in Crs, an important fact considering that small increases in Crs have serious repercussion on the prognosis of patients. Efforts directed to stratify the risk of acute kidney injury (AKI) have crystallized in the RIFLE or AKIN systems, based in sequential changes in Crs or urine flow. These systems have provided a common definition of AKI and, due to their sensitivity, have meant a considerable advantage for the clinical practice but, on the other side, have introduced an uncertainty in clinical research because of potentially overestimating AKI incidence. Another significant drawback is the unavoidable period of time needed before a patient is classified, and this is perhaps the problem to be overcome in the near future.

## 1. Epidemiology of Acute Kidney Injury in the ICU

Acute kidney injury (AKI) can be defined as a decrease of the glomerular filtration rate (GFR) that appears acutely, is maintained for some time, causes an accumulation of waste products from metabolism and uremic toxins, and conditions a mishandling of body fluids and a loss of the ability to maintain homeostasis of electrolytes and acid-base balance. In the intensive care setting, AKI presents with a high incidence and, once established, has an important impact in the patient and the resources [1, 2].

The reported incidence of AKI in the intensive care units (ICUs) shows a wide variability depending on the population analyzed and the criteria employed for its definition, but when this is based in the new systems for stratification, as RIFLE [3] or AKIN [4], more than 30% of ICU patients are found to present with some degree of AKI [5], and mortality rate increases according to this degree of renal dysfunction [6, 7]. These figures are a good measure of the magnitude of the problem, and even when functional recovery after AKI is good, it has been demonstrated that the development of severe AKI can lead to an increase in long-term mortality [8] with an estimation of the incidence of chronic kidney disease (CKD) after an episode of AKI as high as 7.8 per 100 patients per year [9, 10].

This scenario has put in evidence the necessity of new tools for continuous assessment of kidney function given that the classical approach of measurements of isolated determinations of serum creatinine (Crs) has proven insufficient [11].

## 2. Measuring Kidney Function

### 2.1. Glomerular Filtration Rate

**Figure 1:**
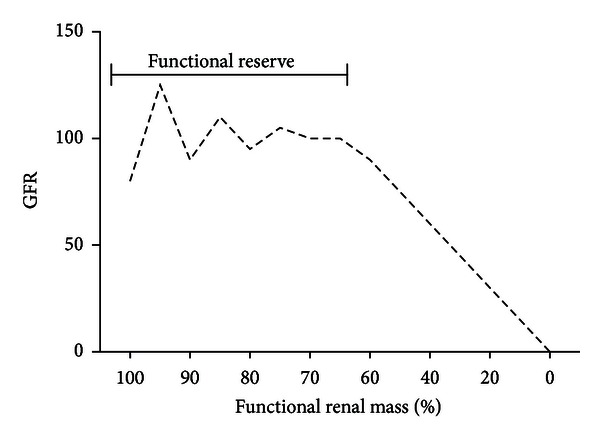
When enough renal mass is present, GFR changes in response to patient necessities, but when damage is severe this “renal reserve” is lost. Adapted from [12].

One way to evaluate renal function is studying its capability to maintain an adequate rate of filtration in the glomerulus, that is, the GFR. The GFR is a measure of the amount of blood filtered per unit of time but not necessarily of kidney damage. We must keep in mind that a direct relationship between renal mass and changes in GFR does not exist until late in the process of damage because the kidneys are able to compensate the loss of renal mass through a raise in the filtration rate in those nephrons still functioning (Figure 1) [12]. GFR can be estimated by the measurement of the rate of elimination (clearance) of different molecules that are filtered by the glomerulus but not secreted or reabsorbed by the tubule, and the use of endogenous molecules naturally producing in the organism has been proposed for this purpose, mainly Crs.

### 2.2. Serum Creatinine

**Figure 2:**
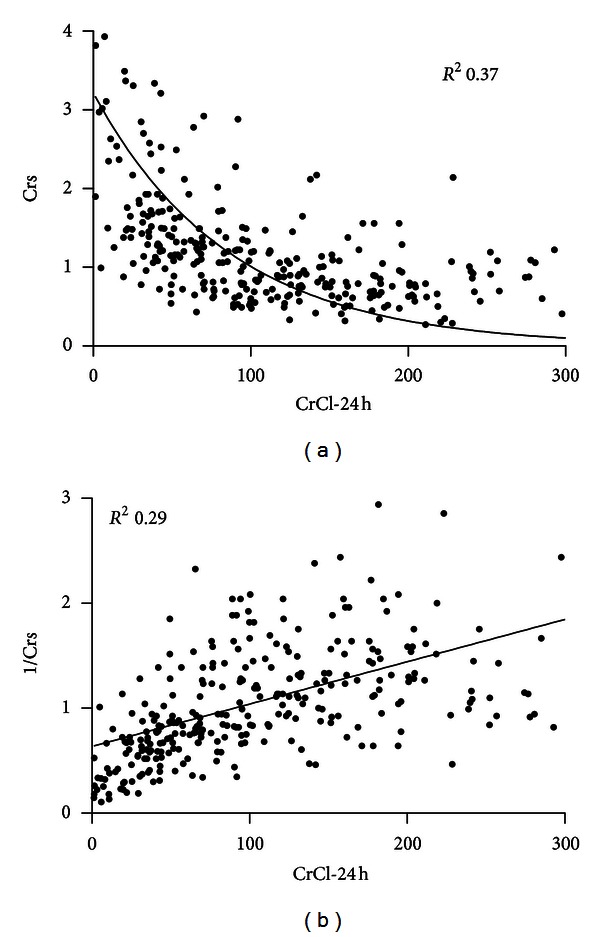
Relationship between serum creatinine and creatinine clearance. Data from the authors, adapted from [13].

Crs is an organic protein resulting from the degradation of creatine, produced and eliminated at a constant rate, exclusively cleared by the kidneys, and filtered at the glomerulus without significant tubular reabsorption or tubular secretion. Its main drawback is based on the fact that changes in Crs do not follow a linear relationship with changes in GFR, so that when detecting changes in its concentration we must not assume similar changes in the GFR (Figure 2) [16]. Also, Crs being an endogenous molecule, its metabolism is subjected to interpersonal variations depending on different factors [17]. Taking into consideration these aspects, Crs is still the parameter universally adopted for the diagnosis of kidney failure, but we must keep in mind that its value reflects the functionality of the kidney and not necessarily the presence of actual damage. 

**Figure 3:**
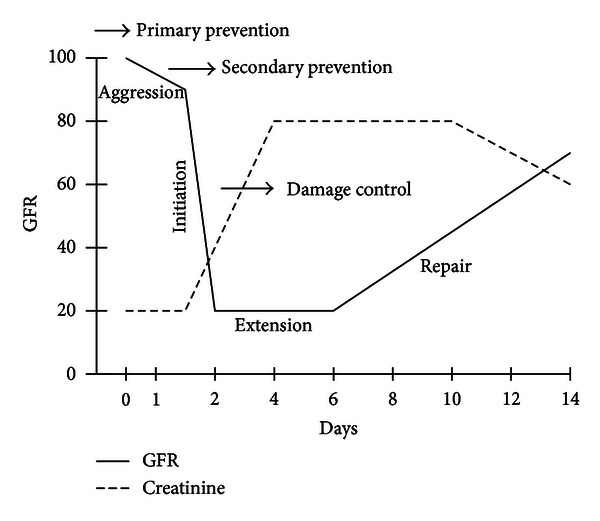
Relationship between glomerular filtration rate (GFR) and serum creatinine (Crs) in time. Adapted from [14, 15].

Crs is a functional parameter and its role in the diagnosis of renal injury is closely related to what we can address as “renal reserve.” When a patient initially presenting a normal Crs concentration surpasses 2 mg/dL, he or she may have lost approximately 50% of the functioning renal mass [12, 14], but on the other hand, changes in Crs after a serious renal insult depend largely on the basal figures as well, so that 24 hours after a 90% fall in the creatinine clearance (CrCl), the increase of Crs might be up to 246% when kidney function is normal but only 174% when the patient already featured a dysfunction in stage 2 of the KDIGO guidelines [18] or as low as 74% when the patient was in stage 4, for a virtually identical absolute increase in the Crs (between 1.8 and 2 mg/dL). For this reason, some authors have advocated for a definition of AKI based upon changes of Crs levels for a given period (between 24 and 48 hours) instead of absolute serum levels [19]. This approach palliates the problem derived from the delayed raise in Crs (more than 48 hours) following a change in GFR (Figure 3) [14, 15, 18, 19].

### 2.3. Fluids and Crs

Another key point when assessing serial changes in Crs is the repercussion of the balance of fluids. In those situations when aggressive hydration has been necessary and water balance is positive, the relative serum concentration of Crs decreases and therefore underestimates the real value [20–23].

### 2.4. Crs Assay

The method described by Jaffé for the assay of Crs has been the cornerstone for the diagnosis of the renal failure until recently but shows variations for a range from 0.06 to 0.31 mg/dL, a range previously considered safe but is now considered to be of potential prognostic value [24, 25]. This fact has favored its displacement by the enzymatic assay [26].

### 2.5. Creatinine Clearance

This method does in effect show a lineal relationship with GFR and is less affected by the delayed changes of Crs after GFR decrement but shares all the other problems of the Crs already mentioned. In routine ICU clinical practice, CrCl measured with diuresis of 24 hours is not operational, and different investigators have sought alternatives more adapted to the ICU environment. An approach is the measurement of CrCl with samples of urine collected in shorter intervals of time, making repetitive measures more feasible, urine samples from simultaneous patients easier to handle, and (the most critical aspect) without delay for the results [27]. Different timings for collection of urine have been validated by some authors, ranging from one hour by Hoste et al. [28], two hours by Herrera-Gutiérrez et al. [13] or periods from two to twelve hours by Wilson and Soullier [29]. In addition, these studies show how among those patients with Crs in normal or near-normal range (below 1.5 mg/dL) up to 25% already had a significantly diminished CrCl and also put in evidence that equations for estimation of GFR in the ICU (Cockroft-Gault and MDRD) are not adequate [13, 28]. However, despite the scarcity of studies addressing the validity of these equations in the acute patient (and specifically in the ICU patient) and the general agreement against its use in this scenario, these equations (especially MDRD) have become the usual tool for estimation of CrCl and guiding prescription of drugs that require adjustment in the presence of renal dysfunction [30, 31]. When an exact measure is deemed necessary none of these equations replaces a measurement of CrCl [32]. 

### 2.6. Cys-C

Cys-C is a low molecular weight protein produced by all nucleated cells at a constant rate, being filtered by the glomerulus and reabsorbed and metabolized in the proximal tubule without tubular secretion and only minimal extrarenal elimination. Cys-C has shown promising results as an estimator of GFR in patients with stable renal function [33, 34]. 

### 2.7. Biomarkers of Kidney Injury

Different biomarkers of kidney injury have recently been evaluated with mixed results [35]. It is still necessary to define the kinetics of these molecules and their relationship to the development of kidney injury [36]. Another important point to emphasize is that these new biomarkers are not aimed to the assessment of renal function (do not estimate GFR) and therefore can not replace but are complementary to Crs or Cys-C. 

## 3. Stratification of AKI

**Figure 4:**
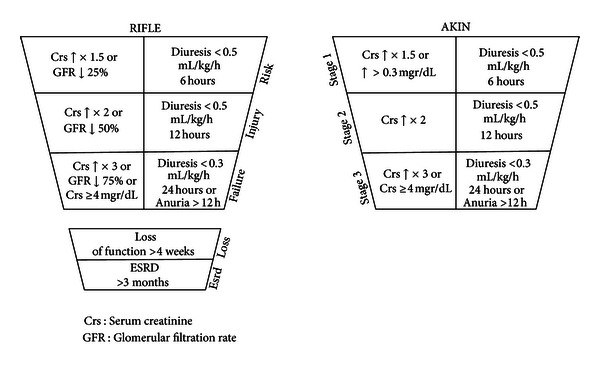
RIFLE and AKIN classification systems [3, 4].

From the moment the aggression occurs until the kidneys begin to show alterations and dysfunction, different mechanisms of compensation have been launched that which produce a decrease in the GFR [37] but, due to the lack of sensible methods of diagnosis, we acknowledge the presence of this renal failure once this initial phase has already been surpassed. This problem is worsened because there is not a clear definition of what we must consider AKI [38, 39] but the definition of two systems aimed to stratify acute kidney dysfunction based on sequential changes of Crs (RIFLE and AKIN) has come to fill this gap for the AKI patient (Figure 4) [3, 4].

The RIFLE (an acronym for risk, injury failure, loss, and end-stage) system made a proposal for a new definition considering AKI as a dynamic process. Another major advantage of this system was its simplicity, advocating for the use of biomarkers universally affordable (Crs and diuresis). In 2007, the AKIN (acute kidney injury network) group designed a new stratification system based on the premises of the RIFLE system but incorporating the findings from Lassnigg et al. that demonstrated how small increases in Crs carry a proportional increase in mortality [24, 40–43]. These two systems have been evaluated in large series of UCI patients and are currently consolidated as reference, but both systems present some problems [44] and their introduction has conditioned a substantial increase in the incidence of AKI published, having in fact increased on the order of 2 to 10 times [45].

The problem, shared by both systems, is the need for a minimum timeframe to proceed with the classification, which in RIFLE extends up to a week and in AKIN for 48 hours. This inevitable time window will condition a delay in the detection of AKI. Yet another problem with RIFLE arises from the possibility to choose indistinctly between CrCl or Crs, even when these values are not linearly related and do not change simultaneously in time [46]. Another relevant aspect and one that questions the consistency of these systems is the finding of similar outcomes for patients in the AKIN 1 and 2 levels with a significant increment for level 3 and with a similar behavior for RIFLE [47] that could be suggesting the convenience for a reappraisal of the ranges of Crs considered in each level.

## 4. Conclusion

A proper definition for AKI should establish the presence or absence of the disease, report on its severity and prognosis, and, perhaps more important, should be easy to understand and implement [48]. Although these assumptions have been partly met by RIFLE and AKIN, it is likely that in a near future our understanding about AKI and its impact will be modified. 

It is important to stress the fact that at least 20% of hospitalized patients develop some degree of renal dysfunction, and the prognosis for these patients worsens as the degree of dysfunction increases [49] but the fact is that for those who survive, only 10% will eventually be in need for prolonged renal replacement [50]. These figures reinforce the relevance of a timely detection of impending AKI in order to apply secondary preventive measures and limit its progression, increasing the chances of recovery of our patients.

Although Crs is a parameter sensible for deciding whether a patient's kidney function remains stable, worsens, or improves, its role in the diagnosis of early renal dysfunction is more debatable, and in order to evaluate the information it provides we must understand the pathophysiology of acute renal failure and the kinetics of creatinine (be it Crs, measured CrCl or estimated by equations), and in any case, we must integrate this information in one of the stratification systems currently in use, but always acknowledging their limitations. 

Serum creatinine is the key factor in the evaluation of kidney function because it is affordable, reproducible, and easy to perform, but clinicians must be aware of its limitations, among others that it is a functional marker and not a marker of injury, that changes in Crs are delayed after changes in GFR, or that fluid changes in critically ill patients can seriously difficult the capability of Crs to detect small changes in kidney function. 

New trends in stratification (ADQI or AKIN) could have a significant impact in clinical practice, alerting the clinicians of the real value of small changes in Crs, and the novel biomarkers of kidney damage (in particular of tubular injury) may in the near future have a role in the diagnosis of AKI once they are included in the classification systems.

## References

[1] Joannidis M, Metnitz PGH (2005). Epidemiology and natural history of acute renal failure in the ICU. *Critical Care Clinics*.

[2] Uchino S, Kellum JA, Bellomo R (2005). Acute renal failure in critically ill patients: a multinational, multicenter study. *Journal of the American Medical Association*.

[3] Bellomo R, Ronco C, Kellum JA, Mehta RL, Palevsky P (2004). Acute renal failure—definition, outcome measures, animal models, fluid therapy and information technology needs: the second international consensus conference of the acute dialysis quality initiative (ADQI) group. *Critical Care*.

[4] Mehta RL, Kellum JA, Shah SV (2007). Acute kidney injury network: report of an initiative to improve outcomes in acute kidney injury. *Critical Care*.

[5] Ostermann M, Chang RWS (2007). Acute kidney injury in the intensive care unit according to RIFLE. *Critical Care Medicine*.

[6] Hoste EAJ, Clermont G, Kersten A (2006). RIFLE criteria for acute kidney injury are associated with hospital mortality in critically ill patients: a cohort analysis. *Critical Care*.

[7] Ricci Z, Cruz D, Ronco C (2008). The RIFLE criteria and mortality in acute kidney injury: a systematic review. *Kidney International*.

[8] Schiffl H, Fischer R (2008). Five-year outcomes of severe acute kidney injury requiring renal replacement therapy. *Nephrology Dialysis Transplantation*.

[9] Murugan R, Kellum JA (2011). Acute kidney injury: what’s the prognosis?. *Nature Reviews Nephrology*.

[10] Coca SG, Yusuf B, Shlipak MG, Garg AX, Parikh CR (2009). Long-term risk of mortality and other adverse outcomes after acute kidney injury: a systematic review and meta-analysis. *American Journal of Kidney Diseases*.

[11] Dennen P, Douglas IS, Anderson R (2010). Acute kidney injury in the intensive care unit: an update and primer for the intensivist. *Critical Care Medicine*.

[12] Bellomo R, Kellum JA, Ronco C (2004). Defining acute renal failure: physiological principles. *Intensive Care Medicine*.

[13] Herrera-Gutiérrez ME, Seller-Pérez G, Banderas-Bravo E, Muñoz-Bono J, Lebrón-Gallardo M, Fernandez-Ortega JF (2007). Replacement of 24-h creatinine clearance by 2-h creatinine clearance in intensive care unit patients: a single-center study. *Intensive Care Medicine*.

[14] Moran SM, Myers BD (1985). Course of acute renal failure studied by a model of creatinine kinetics. *Kidney International*.

[15] Liu KD (2003). Molecular mechanisms of recovery from acute renal failure. *Critical Care Medicine*.

[16] Stevens LA, Coresh J, Greene T, Levey AS (2006). Assessing kidney function—measured and estimated glomerular filtration rate. *The New England Journal of Medicine*.

[17] Beddhu S, Samore MH, Roberts MS, Stoddard GJ, Pappas LM, Cheung AK (2003). Creatinine production, nutrition, and glomerular filtration rate estimation. *Journal of the American Society of Nephrology*.

[18] Levey AS, Eckardt KU, Tsukamoto Y (2005). Definition and classification of chronic kidney disease: a position statement from kidney disease: improving global outcomes (KDIGO). *Kidney International*.

[19] Waikar SS, Bonventre JV (2009). Creatinine kinetics and the definition of acute kidney injury. *Journal of the American Society of Nephrology*.

[20] Macedo E, Bouchard J, Soroko SH (2010). Fluid accumulation, recognition and staging of acute kidney injury in critically-ill patients. *Critical Care*.

[21] Liu KD, Thompson BT, Ancukiewicz M (2011). Acute kidney injury in patients with acute lung injury: impact of fluid accumulation on classification of acute kidney injury and associated outcomes. *Critical Care Medicine*.

[22] Bouchard J, Mehta RL (2009). Fluid accumulation and acute kidney injury: consequence or cause. *Current Opinion in Critical Care*.

[23] Prowle JR, Echeverri JE, Ligabo EV, Ronco C, Bellomo R (2010). Fluid balance and acute kidney injury. *Nature Reviews Nephrology*.

[24] Lassnigg A, Schmidlin D, Mouhieddine M (2004). Minimal changes of serum creatinine predict prognosis in patients after cardiothoracic surgery: a prospective cohort study. *Journal of the American Society of Nephrology*.

[25] Udy A, O’Donoghue S, D’Intini V, Healy H, Lipman J (2009). Point of care measurement of plasma creatinine in critically ill patients with acute kidney injury. *Anaesthesia*.

[26] Myers GL, Miller WG, Coresh J (2006). Recommendations for improving serum creatinine measurement: a report from the laboratory working group of the national kidney disease education program. *Clinical Chemistry*.

[27] Lameire N, Hoste E (2004). Reflections on the definition, classification, and diagnostic evaluation of acute renal failure. *Current Opinion in Critical Care*.

[28] Hoste EAJ, Damen J, Vanholder RC (2005). Assessment of renal function in recently admitted critically ill patients with normal serum creatinine. *Nephrology Dialysis Transplantation*.

[29] Wilson RF, Soullier G (1980). The validity of two-hour creatinine clearance studies in critically ill patients. *Critical Care Medicine*.

[30] Lameire N, Adam A, Becker CR (2006). Baseline Renal Function Screening. *American Journal of Cardiology*.

[31] Herrera-Gutiérrez ME, Seller-Pérez G, Maynar-Moliner J,  Sánchez-Izquierdo Riera JA (2012). Variability in renal dysfunction defining criteria and detection methods in intensive care units: are the international consensus criteria used for diagnosing renal dysfunction?. *Medicina Intensiva*.

[32] Seller-Pérez G, Herrera-Gutiérrez ME, Banderas-Bravo E, Olalla-Sánchez R, Lozano-Sáez R, Quesada-García G (2010). Concordance in critical patients between the equations designed for the calculation of glomerular filtration rate and 24-hour creatinine clearance. *Medicina Intensiva*.

[33] Nejat M, Pickering JW, Walker RJ, Endre ZH (2010). Rapid detection of acute kidney injury by plasma cystatin C in the intensive care unit. *Nephrology Dialysis Transplantation*.

[34] Herget-Rosenthal S, Bökenkamp A, Hofmann W (2007). How to estimate GFR-serum creatinine, serum cystatin C or equations?. *Clinical Biochemistry*.

[35] Coca SG, Yalavarthy R, Concato J, Parikh CR (2008). Biomarkers for the diagnosis and risk stratification of acute kidney injury: a systematic review. *Kidney International*.

[36] Endre ZH, Pickering JW, Walker RJ (2011). Improved performance of urinary biomarkers of acute kidney injury in the critically ill by stratification for injury duration and baseline renal function. *Kidney International*.

[37] Lameire N, Van Biesen W, Vanholder R (2005). Acute renal failure. *Lancet*.

[38] Mehta RL, Chertow GM (2003). Acute renal failure definitions and classification: time for change?. *Journal of the American Society of Nephrology*.

[39] Bellomo R, Kellum J, Ronco C (2001). Acute renal failure: time for consensus. *Intensive Care Medicine*.

[40] Barrantes F, Tian J, Vazquez R, Amoateng-Adjepong Y, Manthous CA (2008). Acute kidney injury criteria predict outcomes of critically ill patients. *Critical Care Medicine*.

[41] Chertow GM, Burdick E, Honour M, Bonventre JV, Bates DW (2005). Acute kidney injury, mortality, length of stay, and costs in hospitalized patients. *Journal of the American Society of Nephrology*.

[42] Thakar CV, Christianson A, Freyberg R, Almenoff P, Render ML (2009). Incidence and outcomes of acute kidney injury in intensive care units: a veterans administration study. *Critical Care Medicine*.

[43] Coca SG, Peixoto AJ, Garg AX, Krumholz HM, Parikh CR (2007). The prognostic importance of a small acute decrement in kidney function in hospitalized patients: a systematic review and meta-analysis. *American Journal of Kidney Diseases*.

[44] Cruz DN, Ricci Z, Ronco C (2009). Clinical review: RIFLE and AKIN—time for reappraisal. *Critical Care*.

[45] Hoste EA, Kellum JA (2006). Acute kidney injury: epidemiology and diagnostic criteria. *Current Opinion in Critical Care*.

[46] Herrera-Gutiérrez ME, Seller-Pérez G, Banderas-Bravo E, Aragón-Gonzalez C, Olalla-Sánchez R, Lozano-Sáez R (2011). Discrepancies in the rifle classification are due to the method used to assess the level of derangement of kidney function. *Journal of Critical Care*.

[47] Mandelbaum T, Scott DJ, Lee J (2011). Outcome of critically ill patients with acute kidney injury using the acute kidney injury network criteria. *Critical Care Medicine*.

[48] Ricci Z, Cruz DN, Ronco C (2011). Classification and staging of acute kidney injury: beyond the RIFLE and AKIN criteria. *Nature Reviews Nephrology*.

[49] Uchino S, Bellomo R, Goldsmith D, Bates S, Ronco C (2006). An assessment of the RIFLE criteria for acute renal failure in hospitalized patients. *Critical Care Medicine*.

[50] Morgera S, Schneider M, Neumayer HH (2008). Long-term outcomes after acute kidney injury. *Critical Care Medicine*.

